# Evidences for better recall of congruent items in episodic memory

**DOI:** 10.1007/s10339-020-00963-x

**Published:** 2020-02-21

**Authors:** X. Laurent, A. F. Estévez, P. Marí-Beffa

**Affiliations:** 1 University of Oxford, Oxford, UK; 2 University of Almería, Almería, Spain; 3 University of Bangor, Bangor, UK

**Keywords:** Episodic memory, Selective attention, Priming, Congruency

## Abstract

A focus of recent research is to understand the role of our own response goals in the selection of information that will be encoded in episodic memory. For example, if we respond to a target in the presence of distractors, an important aspect under study is whether the distractor and the target share a common response (congruent) or not (incongruent). Some studies have found that congruent objects tend to be grouped together and stored in episodic memory, whereas other studies found that targets in the presence of incongruent distractors are remembered better. Our current research seems to support both views. We used a Tulving-based definition of episodic memory to differentiate memory from episodic and non-episodic traces. In this task, participants first had to classify a blue object as human or animal (target) which appeared in the presence of a green one (distractor) that could belong to the same category as the target (congruent); to the opposite one (incongruent); or to an irrelevant one (neutral). Later they had to report the identity (What), location (Where) and time (When) of both target objects (which had been previously responded to) and distractors (which had been ignored). Episodic memory was inferred when the three scene properties (identity, location and time) were correct. The measure of non-episodic memory consisted of those trials in which the identity was correctly remembered, but not the location or time. Our results show that episodic memory for congruent stimuli is significantly superior to that for incongruent ones. In sharp contrast, non-episodic measures found superior memory for targets in the presence of incongruent distractors. Our results demonstrate that response compatibility affects the encoding of episodic and non-episodic memory traces in different ways.

## Introduction

Humans seem to be very good at remembering a great deal of information from specific episodes in their lives. For example, if someone asks what you had for breakfast this morning, you may remember everything you ate, the position of the different pieces of food on the table, and even the order in which you ate them. The grouping of all these pieces of information in long-term memory has been studied as part of the episodic memory system (Tulving 1972). Recently, there is increasing evidence that the way we interact with various objects also influences this type of memory. Indeed, previous experiments from our laboratory (Laurent et al. 2015) demonstrated that the action provokes the suppression of irrelevant distractor encoding in episodic memory, therefore remembering better those objects that were relevant to our task goals. Following the previous example, even if you did not eat the black pudding, being a potential breakfast item will make it better remembered than a pencil placed in the same location on the table. Our conscious experience of the world, therefore, may not be limited to information that we paid attention to and interacted with, but it may also involve irrelevant objects or contextual aspects of the scene that are bound together to form an episodic trace (Frings et al. 2013).

In this sense, some authors suggest that objects in a scene tend to group together if they are part of a set, leaving out other objects not belonging to the set (Gollin and Sharps 1988). This goal-related grouping or ‘chunking’ (Miller 1956) has the advantage of flexibly increasing or reducing the amount of information we store, as long as it is related to the task at hand (Baddeley et al. 1984). This view has received support from studies which found that response compatibility enhances episodic memory. Using the Attentional Network Test, some authors have recently found that response compatible items during encoding were more easily bound to their contexts than incompatible ones. These benefits were observed in a subsequent recognition test that analyzed memory of the object, of the spatial location and of the condition (compatible/incompatible) in which it appeared (Sperduti et al. 2017). These results are interesting because they reveal that binding processes taking place in short-term memory but due to attentional mechanisms they can have long-term memory effects. In this article, we explore the possibility of these long-term effects having a specific influence in episodic memory.

One interesting example of this relationship between goal-directed attention and episodic memory is the work of Moeller and Frings (2014a). They wondered whether the binding of responses to targets extends to distractors. In their view, attending to one part of the scene brings together other aspects of it, especially those related to the target object. This concept of short-term episodic trace is based on the attentional grouping of perceptual features (object files; Kahneman et al. 1992) and even responses (event files; Hommel et al. 2001; Logan 1988) within an object; that is, attending to one part of an object induces processing of the whole object (Baylis and Driver 1992). When responding to a target in a scene, co-occurring distractors can also be integrated with responses and be retrieved later when an associated response is required (the distractor–response binding effect; Frings 2011; Frings and Möller 2010). This distractor binding, however, seems to emerge in conditions where the distractor information is somehow relevant for the task. When distractor information is completely irrelevant, this binding with the target may not occur (Logie 1995; Del Gatto et al. 2016; note also that differences in the nature of the task can also influence the grouping of specific irrelevant properties, Delogu et al. 2012; Jaswal 2013). Under this perspective, relevant distractors may receive increased attention, not being selectively segregated from the target and therefore encoded together with it as part of the same episodic event (Moeller and Frings 2014a).

Additional evidence for this response grouping within episodic events emerges from studies finding that responses to an object that was previously presented as a compatible distractor shows enhanced performance (facilitation of responses in the response binding effect) compared to incongruent ones (Moeller and Frings 2014b). When the distractor was strongly associated to the same response required for the target (e.g., an arrow pointing right or left for a right/left response), the distractor response binding effect was greater than in any other condition. Although these authors made no explicit connection between these short-term memory traces and the encoding in long-term episodic memory, the possible relation between these two processes is at the core of our study.

On the contrary, a different theoretical perspective suggests that episodic memory improves when an object appears in the presence of conflicting, rather than congruent, information (Rosner et al. 2015). The logic behind this perspective is that the presence of conflict triggers an increase in selective attention (to resolve the conflict), with the subsequent impact on episodic memory encoding. It is important to highlight that this view makes no predictions about the encoding of distractor information; instead it predicts the indirect impact of these distractor objects on the episodic encoding of selected targets. The role of the distractor, in this view, is only to generate conflict during encoding, but it does not need to be represented as part of the full episodic trace. Accordingly, in their study, Rosner et al. (2015) asked whether congruency between distractor and target words at the time of encoding influences episodic memory. They presented two interleaved words written in different colors (target in red and distractor in green), and the task was to name the target word. The distractor word could be identical to the target one (congruent) or different (incongruent). They found that recognition memory for incongruent targets was better than for congruent ones.

Importantly, despite the interest on episodic learning, the grouping of different elements of episodic memory was never tested in the study by Rosner et al. (2015). Indeed, they did not measure memory of any other aspects of the episodic trace, such as space or time. Even more importantly, they collected data on recognition memory only from the target and never from the distractors. They found a boost in recognition memory for targets only in the incongruent condition, concluding that there is a link between cognitive control (via selective attention) and episodic memory.

Our position in this article is that without testing other elements of the episodic trace, it is impossible to draw any conclusions about how episodic memory changes in the different congruency conditions. Rosner et al. (2015) used a type of recognition test (old/new) that could be affected by other types of memory that are not necessarily episodic. Arguably, a participant could respond to an ‘old’ object based on a level of implicit or even semantic familiarity that may not require the retrieval of the entire episode in which it appeared. For this reason, their predictions cannot readily be extrapolated to explicit episodic memory tasks. In support of this argument, several studies have found that attentional states can modulate implicit retrieval processes without influencing explicit ones (Boronat and Logan 1997; Logan and Etherton 1994; Logan et al. 1999).

In the present study, we specifically tested whether explicit episodic retrieval of target and distractor objects would be affected by their response compatibility at the time of encoding. We used a What–Where–When (WWW) task that collects information about object identity (What), spatial location (Where) and temporal order (When) (Laurent et al. 2015). In this task, episodic memory is inferred when all three object features (identity, location and time) are correctly remembered. Memory increases or reductions that may be observed partially for some of these features (e.g., object and place correct, but not the time) are not considered to be episodic. Therefore, if the response grouping theory is correct, then congruent targets and distractors would be better remembered episodically than the incongruent ones. If, on the other hand, the resolution of conflict enhances episodic memory of target objects, incongruent targets should be remembered better episodically than the congruent ones. This theory, as stated by Rosner et al. (2015), makes no clear predictions about memory from distractors, as their role would be to direct attention toward the target resulting from their conflict.

## Methods

### Participants

The preferred sample size was estimated a priori using G*Power 3.1.9.2 (Erdfelder et al. 1996). Setting *α* to 0.05 and a desired power of 0.90 or above for a repeated measures factorial ANOVA (6 repeated measures), we expected to obtain an effect size of 0.25 and repeated measures correlations of 0.2 producing a minimum required sample size of 44.

Fifty-three volunteers (41 females, 12 males)^1^ were recruited to take part in this study; they ranged in age from 18 to 55 years (*M* = 23, SD = 7.2). Incidental and snowball sampling procedures were performed. The tests were conducted at the University of Almeria (in Spanish) with undergraduate psychology students; results were sent to Bangor University for analysis. All participants gave written informed consent before data collection began, but were naïve about the goals of the test. They all received one course credit for their participation. None of the participants reported having any memory deficit or other cognitive dysfunction. They all reported that they have normal or corrected-to-normal vision. The internal ethics committee of the School of Psychology (Bangor University) approved the study.

### Apparatus and stimuli

The objects used for the memory task were all drawn from a string of standardized pictures from the Snodgrass and Vanderwart (1980) database. The 24 pictures (hereafter referred to as ‘objects’) chosen for the congruent, incongruent and neutral conditions were 10 animals (A), 10 humans (H) and 6 other items (N). Twenty-four additional objects (never seen at encoding) were used during the cue recall phase. The stimuli for this task were presented using desktop computers with a 15″ VGA monitor running at a resolution of 1024 × 768 pixels for viewing from about 60 cm. On average, the visual angle for each object was 2.2°. All stimuli appeared on a static screen with a white background, with two objects appearing next to each other.

**Fig. 1:**
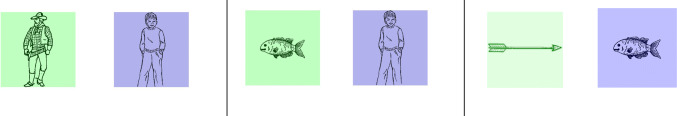
Example of “objects” presented during the encoding phase. The congruent condition (human–human), the incongruent condition (animal–human) and the neutral condition (animal–neutral item)

During the encoding phase, participants received a series of 12 trials occurring in two blocks of six and separated by a cross (×) in the middle of the block. In each trial, participants saw two objects, one blue and one green (Fig. 1). The trial was set up with three conditions, each combining different ‘objects’:

- four trials in the *congruent* condition (2 animal target, animal distractor—AA; and 2 human target, human distractor—HH);
- four trials in the *incongruent* condition (2AH, 2HA); and
- four trials in the *neutral* condition (2AN and 2HN) (Fig. 1).

The 12 trials were randomly presented and consisted of a total of 24 objects^2^ from the Snodgrass and Vanderwart database, presented with no repetition. Target objects were in blue and distractor objects in green; target and distractor objects alternated left and right on the screen for each trial to encourage both full attention to the target spatial location while stimulating at the same time the processing of the entire scene. The participant task was to attend to the blue (ignore the green) and indicate as quickly and accurately as possible if it was a human or animal object. The objects remained on the screen until response, with a maximum response window of 3 s. If the blue object was human, participants pressed key C, if the blue object was an animal, they pressed the M key. Participants knew in advance that there would be a general memory task immediately after these trials but were not told about the specific properties to memorize nor the need to remember a particular type of object (target or distractor).

**Fig. 2:**
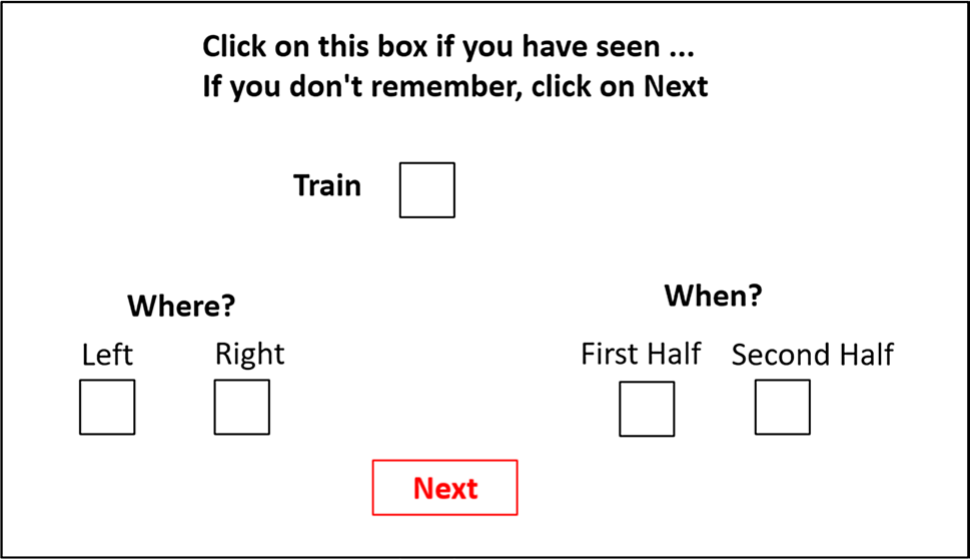
Example of screen (the text has been translated in English for this paper) presented to student during the cue recall phase, with the name of the item to recall (either old or new)

For the cue recall phase (Fig. 2), participants were asked to report whether they remembered any of the objects previously presented (regardless whether they were targets or distractor), according to their identity, spatial position and time in which they appeared. To test for this, they received in successive trials a random presentation of names corresponding to the 24 objects used during the previous phase, along with 24 new object names from the Snodgrass and Vanderwart (1980) database, belonging to the same categories, human/animal/neutral (e.g., soldier, whale, train …). First, for the What task, participants were asked if they remembered the object by presenting the name of the object as a cue at the top of the screen with a tick box next to it. If they did not, then they had to move to the next slide by clicking a ‘Next’ button. However, if they believed that they remembered the object, then they had to click the tick box, which would trigger the Where and When forced choice questions. For the memory of spatial location (Where), there were two tick boxes so the participant had to choose one to represent the spatial location of the object, left or right. For the memory about the temporal position (When), participants saw two other tick boxes to represent whether the object appeared before the mid-block cross or after the cross. The duration of the total task, including the recall phase, was an average of 10 min.

A conservative approach was used, in which episodic retrieval of a particular object was inferred only in those cases where the three memory components (What, Where and When) were correct (a measure of full episodic recall). However, partially recalled objects were also measured for object identity, spatial location and temporal position. We analyzed these as three non-episodic measures of object recall: ‘What’ means that the object was correctly identified, but they failed to report where and when it appeared; ‘Where’ means that both object and location were correctly identified, but they failed to report when it appeared; and ‘When’ means that both object and time where correctly reported, but the location was incorrect. None of these non-episodic objects^3^ are contained in the full measure of Episodic Memory. Each of these types of recall was considered meaningful when its rate appeared significantly above the chance level of 0.125.^4^

## Results

### Encoding

Data from three participants were removed, as their accuracy levels for recall fell in the area of 1.5 IQR above the third quartile. Means of Reaction Times (RTs) per participant per condition were analyzed through a repeated measures ANOVA to test for response compatibility effects across congruent, incongruent and neutral trials. Results demonstrated significant differences among conditions [*F*(2,98) = 5.32; *p* = 0.006; *η*_*p*_^2^ = .098], where responses for congruent trials (RT = 975.86, SD = 235.98) were 76 ms faster than for incongruent ones (RT = 1052,24, SD = 241.65) (*p* = 0.009), and 99 ms faster than neutral ones (RT = 1075.19, SD = 196.25) (*p* = 0.001). Responses between incongruent and neutral trials were not different (*p* = 0.489).

### Retrieval

Responses were separated depending on whether they were given to any of the 24 old items presented during the encoding phase (correct recalls and misses) or to the 24 new items only appearing in the retrieval phase (false alarms and correct rejections). Subsequent analyses were conducted on correct recalls only after computing corresponding accuracy rates for each condition. Overall correct recall rate for all conditions was .41 (SD = .25). For the new items, overall false alarms rate was .12 (SD = .13).

Accuracy rates (proportion of correct responses out of all responses) were then computed from the correct recalls differently for the four memory conditions (Full Episodic Recall, Object Only, Object and Space, Object and Time). Means of accuracy rates for each participant and condition were analyzed through a repeated measures factorial ANOVA for a 2 (objects: target, distractor) × 3 (response congruency: congruent, incongruent, neutral) design. Bonferroni corrections were applied for further comparisons across levels of congruency.

#### Full episodic recall

In this analysis, we calculated accuracy rates for those responses in which participants correctly remembered the object, the position and the moment when it was presented (What, Where and When responses). Following the 2 (Target, Distractor) × 3 (Congruent, Incongruent, Neutral) ANOVA described earlier, results demonstrated that overall recall for targets (*X* = .16, SD = .15) was better than for distractors (*X* = .06, SD = .08); [*F*(1,49) = 24.07; *p* < 0.001; *η*_*p*_^2^ = .329]. In addition, response congruency had an overall impact on object recall [*F*(2,98) = 16.53; *p* < 0.001; *η*_*p*_^2^ = .252], with memory for congruent objects (*X* = .17, SD = .17) being generally better than for incongruent (*X* = .10, SD = .11) (*p* = 0.008) and neutral ones (*X* = .05, SD = .08) (*p* < 0.001), but also incongruent items were remembered better than neutral ones (*p* = 0.013). The difference between memory of targets and distractors did not change depending on the response compatibility (*F* < 1). We then tested the equality of these changes using Bayesian Analysis. The estimated Bayes factors (BF01) for the difference between memory for target and distractors across compatible and incompatible conditions were 7:1 times in favor of the Null Hypothesis. BF01 reached 4:1 for the differences between congruent and neutral conditions, and 9:1 when comparing differences between the incompatible and neutral conditions. These results provide substantial evidence for the stability of the differences in episodic memory from target and distractors across the congruency conditions (Jarosz and Wiley 2014).

**Fig. 3:**
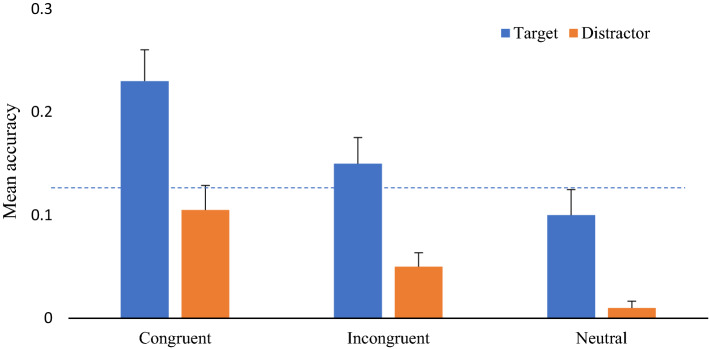
Mean accuracy for full episodic memory. Error bars represent standard errors. The dotted line represents against chance (*p* = 0.125)

Finally, when tested against chance (*p* = 0.125), only congruent targets were significantly remembered above chance (*t*(49) = 3.47, *p* = 0.003), with distractor incongruent and distractor neutral conditions becoming significantly remembered below chance levels (*t*(49) = −5.25, *p* < 0.001, *t*(49) = −16.43, *p* < 0.001, respectively, see Fig. 3).

#### Non-episodic object recall

##### Object only

In this analysis, data represent correct identification of the object with incorrect recollection of the place and moment when it appeared (What correct, Where and When incorrect). This condition provides no evidence of binding of the object with any of the tested contextual properties.

Results again demonstrated better memory for targets (*X* = .19, SD = .18) than for distractors (*X* = .10, SD = .12) [*F*(1,49) = 15.56; *p* < 0.001; *η*_*p*_^2^ = .241]. There were also significant differences due to the compatibility of the responses during encoding [*F*(2,98) = 3.68; *p* = 0.029; *η*_*p*_^2^ = .07]. This time, in sharp contrast to what was found with episodic memory, objects involved in the response incongruent conditions (*X* = .18, SD = .19) were remembered better than in the congruent (*X* = .14, SD = .13; *p* = 0.048) and neutral ones (*X* = .12, SD = .13) *p* = 0.026), with no differences between congruent and neutral responses (*p* = 0.428). There was no interaction between the memory for targets and distractors and the compatibility of responses during encoding (*F* < 1). We further tested the equality of these differences in memory for targets and distractors across the compatibility conditions producing BF01 of 8:1 between compatible and incompatible conditions, 4:1 between compatible and neutral and 7:1 between incompatible and neutral. These results provide substantial evidence that the differences in memory between target and distractors did not change depending on the compatibility during encoding.

**Fig. 4:**
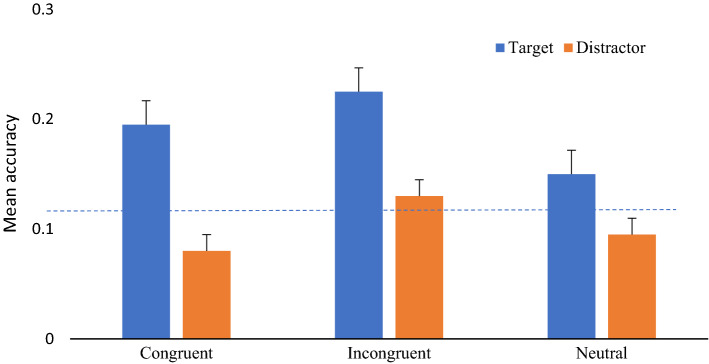
Mean accuracy for object only (What). Error bars represent standard errors. The dotted line represents against chance (*p* = 0.125)

When tested against chance (*p* = 0.125), only target congruent and target incongruent conditions were significant above (*t*(49 = 2.36, *p* = 0.022, t(49) = 2.77, *p* = 0.008, respectively, see Fig. 4).

##### Object and space

For these analyses, we selected only those responses when objects were correctly remembered with the location, but not the time. As mentioned earlier, it is not clear whether these partial bindings are part of episodic memory or not, but they are still reported for information.

Following the previous results, memory for target locations (*X* = .14, SD = .12) was better than for distractors (*X* = .05, SD = .08) [*F*(1,49) = 20.11; *p* < 0.001; *η*_*p*_^2^ = .291], but neither compatibility (*F* < 1) nor interaction (*F* < 1) produced meaningful results. When tested against chance, only memory of target spatial locations was significant (*p* < 0.001).

##### Object and time

Here we report another type of partial binding, referring to correct memory identifications of the time in which an object appeared. Similar to the analysis of spatial partial bindings, we are uncertain about their status as part of the episodic memory trace, presenting them for information.

As observed with spatial representations, memory of the timing of targets (*X* = .08, SD = .09) was superior than for distractors (*X* = .04, SD = .06) [*F*(1,49) = 8.45; *p* = 0.005; *η*_*p*_^2^ = .147]. No other effects were significant. Only memory for target times was above chance (*p* < 0.001).

## Discussion

In this experiment, we aimed to test how the compatibility between target and distractor responses determines the encoding of these events in episodic memory. Existing literature on this issue reveals contradictory evidence. First, some authors have found that response incongruent distractors result in target objects being better represented in episodic memory than their congruent counterparts do (Krebs et al. 2015; Rosner et al. 2015). Those studies focused only on targets and did not test memory of distractors. On the contrary, better episodic memory has been found for congruent objects when compared to incongruent ones (Moeller and Frings 2014b; Sperduti et al. 2017), although distractor and target measures have not always been registered separately. As explained, these two sets of results are commonly obtained from research inspired in two separate theoretical approaches, both differing in the definition and measure of episodic memory. The observation of better memory for congruent objects (Sperduti et al. 2017) generally comes from research considering episodic memory as a long-term memory effect (several minutes) in which multiple features are bound together, similar to the concept that we use in this paper. Conversely, studies finding better memory for incongruent items tend to not measure episodic memory per se, and instead infer a process of episodic retrieval to explain performance in priming tasks. It is possible that these diverging theoretical approaches determine both the methods used to test for episodic memory and the outcomes of these tests.

In this study, we tried to fit our definition of episodic memory as closely as possible to the original one proposed by Tulving (1972), which considers the binding of different types of information in a spatiotemporal context. Therefore, when measuring memory, we considered a retrieval to be ‘episodic’ only when participants accurately remembered the object, its location and when it appeared (What–Where–When test). We also presented partial binding results but because there could be some doubt as to whether these partial binding conditions can be considered episodic or not, we do not discuss them in detail. Using this test, we found that all objects linked to the same response in congruent trials (both target and distractors) were better remembered episodically than for incongruent and neutral displays. The better memory for congruent displays cannot be attributed to a greater time dedicated to encode these scenes, as response times here were significantly shorter than for incongruent displays.

We interpret these results in line with theoretical accounts on attention and object binding put forward by Frings and Möller (2010). According to those authors, when acting on a target object in a scene, co-occurring distractors are likely to be integrated with responses as part of the episodic trace. In this view, the response would open the attentional processing gate, allowing contextual information to be consciously processed and stored. In our case, the congruent response acts as a binding mechanism that improves not only the processing of the target object, but also of the distractors. Our results are in line with the increased distractor response binding effect observed for strong response congruent distractors compared to incongruent ones (Moeller and Frings 2014b). Although no explicit connection was previously made between these short-term binding effects and the encoding in long-term episodic memory, our data seem to support this idea.

Importantly, objects in incongruent trials were remembered better episodically than those in neutral ones. Indeed, memory of targets surrounded by response relevant distractors (whether congruent or incongruent) was superior to the neutral, and response irrelevant, displays. It is also important to note that neither incongruent nor neutral distractors were remembered above chance levels and instead, they were significantly remembered below chance, possibly indicating an interesting influence of distractor inhibition on episodic retrieval (Grison et al. 2005; Laurent et al. 2015; Neill et al. 1992). Thus, any benefit on episodic memory from incongruent responses appears to affect targets exclusively. In conclusion, those targets appearing with response relevant distractors are remembered episodically better than in the presence of neutral ones. This improved memory for congruent and incongruent displays compared to neutral ones has also been found in other episodic memory studies, linking it to the influence of the congruency-dependent interaction between the medial temporal lobe and the medial prefrontal cortex in episodic learning (Van Kesteren et al. 2012). As previously explained, action systems are critical for the formation of episodic events and our data clearly support this claim (Hommel 2010; Moeller and Frings 2014a; Rosner et al. 2015).

In sharp contrast are our results from the non-episodic measures of object encoding. These were found in trials in which participants correctly recognized the object but failed to remember the time or the location. Here we found that memory of incongruent objects was superior to that of congruent and neutral ones. The increased memory of incongruent objects was particularly evident in the case of distractors, as congruent and incongruent targets showed similarly high memory levels, possibly due to some ceiling performance. This pattern strikingly resembles that predicted by Rosner et al. (2015) episodic retrieval account, although in their study target and distractors were indistinguishable. As mentioned earlier, they used priming measures without a specific measure of binding. We suggest that their results reflect the functioning of similar mechanisms and processes to those tested here as non-episodic.

It is possible that our understanding of episodic memory is very different from the one commonly used in attentional research, which inspired Rosner et al. (2015) work (Neill et al. 1992; Neill and Westberry 1987; Mayr et al. 2018). In this field, the term ‘episodic’ is generally used theoretically, to argue against inhibition or to allude to long-term memory processes taking place in selective attention tasks, without a direct measure of it. In addition, it is important to note that an aspect influencing episodic retrieval is the distinctiveness of the episode in which the object appears (Hunt and McDaniel 1993). We can retrieve extremely detailed autobiographical episodes when presented with items unique to that event (such as in the famous Proust ‘madeleine moment’). But when these items appear multiple times in different contexts, each individual episode becomes blurred and less distinctive. In priming tasks, a number of objects appear multiple times in a random order through a large number of trials. In this situation, each individual episode would not be distinctive enough to be retrieved episodically (Frings et al. 2007). In our study, we kept the number of trials to a minimum of six per block, increasing the chances of capturing the impact of episodic retrieval. If this interpretation is correct, then distinctive episodes should increase episodic memory from congruent objects, while less distinctive ones would increase it for the incongruent ones. Future research is required to test this hypothesis.

To sum up, this article provides substantial evidence that the contents of our episodic memory contain both information that was relevant for our tasks at the moment of encoding (information about targets) and information about other objects co-present in the scene that were congruent with our task goals. Previous research in attentional binding already found that congruent objects are bound together in short-term episodic traces (Moeller and Frings 2014b), indicating that response congruency of stimuli leads to grouping of these. Our results have demonstrated that these effects survive several minutes and are able to be retrieved in long-term episodic memory.

## Appendix

See Table 1.

**Table 1:** Objects presented at encoding

Human (10)	Animal (10)	Neutral (4)
Cowboy	Lama	Milk
Fireman	Fish	Backpack
Baby	Ladybug	Arrow
Doctor	Bat	Fishtank
Girl	Beetle	
Nun	Lion	
Dentist	Camel	
King	Crab	
Boy	Elephant	
Beard	Bird	

## Footnotes

1 Because our sample is not balanced, we acknowledge that there are differences in episodic memory with respect to aging (Titz and Verhaeghen 2010). Moreover, there are also gender differences in episodic memory (e.g., Herlitz et al. 1997). Our sample here is not big enough to analyze these potential contributions to our effects, so they need to be addressed in future replications.

2 See “Appendix”.

3 We acknowledge that partial bindings can be also considered episodic to some extent. Rather than classifying these memories as episodic/non-episodic, episodic encoding could be seen as a continuum with different levels of binding. We analyze them separately and will focus the discussion mainly on the results observed in the most extreme conditions: Full Episodic (all properties correctly retrieved) and What (no binding detected).

4 The value of 0.125 is the probability of correctly guessing the three properties—what: 0.5; where: 0.5; when: 0.5.
